# Fragile X mental retardation protein knockdown in the developing *Xenopus* tadpole optic tectum results in enhanced feedforward inhibition and behavioral deficits

**DOI:** 10.1186/s13064-016-0069-7

**Published:** 2016-08-08

**Authors:** Torrey L. S. Truszkowski, Eric J. James, Mashfiq Hasan, Tyler J. Wishard, Zhenyu Liu, Kara G. Pratt, Hollis T. Cline, Carlos D. Aizenman

**Affiliations:** 1 Department of Neuroscience, Brown University, Box G-LN, 60 Olive St., 02912 Providence, RI USA; 2 Department of Molecular and Cellular Neuroscience, Scripps Research Institute, 10550 North Torrey Pines Road, 92037 La Jolla, CA USA; 3 Department of Zoology and Physiology, University of Wyoming, 82071 Laramie, WY USA

**Keywords:** Fragile X syndrome, Fragile X Mental Retardation Protein, inhibition, *Xenopus laevis*, FMRP

## Abstract

**Background:**

Fragile X Syndrome is the leading monogenetic cause of autism and most common form of intellectual disability. Previous studies have implicated changes in dendritic spine architecture as the primary result of loss of Fragile X Mental Retardation Protein (FMRP), but recent work has shown that neural proliferation is decreased and cell death is increased with either loss of FMRP or overexpression of FMRP. The purpose of this study was to investigate the effects of loss of FMRP on behavior and cellular activity.

**Methods:**

We knocked down FMRP expression using morpholino oligos in the optic tectum of *Xenopus laevis* tadpoles and performed a series of behavioral and electrophysiological assays. We investigated visually guided collision avoidance, schooling, and seizure propensity. Using single cell electrophysiology, we assessed intrinsic excitability and synaptic connectivity of tectal neurons.

**Results:**

We found that FMRP knockdown results in decreased swimming speed, reduced schooling behavior and decreased seizure severity. In single cells, we found increased inhibition relative to excitation in response to sensory input.

**Conclusions:**

Our results indicate that the electrophysiological development of single cells in the absence of FMRP is largely unaffected despite the large neural proliferation defect. The changes in behavior are consistent with an increase in inhibition, which could be due to either changes in cell number or altered inhibitory drive, and indicate that FMRP can play a significant role in neural development much earlier than previously thought.

## Background

Fragile X Syndrome (FXS) is the leading monogenetic cause of autism and most common form of inherited intellectual disability [1–3]. FXS is typically caused by the expansion of a trinucleotide (CGG) repeat in the 5′ untranslated region of the Fragile X mental retardation 1 (FMR1) gene [4, 5]. The mutation prevents expression of Fragile X Mental Retardation Protein (FMRP) throughout development. The most well understood neuroanatomical marker in FXS is the presence of immature dendritic spines in the cortex [6, 7]. This is thought to occur because FMRP is a RNA binding protein that inhibits protein synthesis downstream of group 1 metabotropic glutamate receptor activation [8], and therefore prevents normal plasticity and synaptic maturation. However, FMRP disruption prior to synapse formation results in abnormalities that may lead to neurodevelopmental deficits [9], indicating a possible role for FMRP much earlier than initially known.

Previous work from Faulkner et al. [10] identified a cell proliferation defect associated with excessive and decreased levels of FMRP. In this study, *Xenopus laevis* tadpoles were used to assess the early effects of FMRP upregulation and knockdown in the optic tectum. Tadpoles express FMRP throughout central nervous system (CNS) development [11, 12], and thus are ideally placed for studying the effects of FMR1 gene disruption during early development. Since tadpole development occurs in the absence of a womb, this experimental animal provides easy access to developmental stages that occur in utero in mammals [13]. The tadpole optic tectum is homologous to the mammalian superior colliculus and is the main sensory processing area in the tadpole [13]. The optic tectum receives direct visual input via retinal ganglion cells and generates outputs that directly inform behavior, and thus can be used to assay the emergence of functional properties of neural circuits during development.

Faulkner et al. [10] showed that FMR1 is expressed in neural progenitor cells that line the brain ventricle and neurons located lateral to the progenitor cells, as well as in puncta throughout the optic tectum. Both knockdown and overexpression of FMRP reduced cell proliferation in the tectum and increased cell death, providing evidence that FMRP is required at tightly controlled levels. Furthermore, Faulkner et al. [10] showed that FMRP regulates neuronal differentiation and dendritic morphology, with both overexpression and knockdown of FMRP levels resulting in abnormal numbers of neural progenitor cells and reduced dendritic arborization of tectal neurons. These results indicate a critical role for FMRP in early development, both in the generation of new neurons and in the wiring of the proper neural circuit. These data also suggest a clear role for FMRP prior to synapse formation. However, this study did not investigate the consequences of abnormal proliferation and arborization on the functional properties of tectal circuits. Furthermore, it is not clear how the cells that do survive are affected by knockdown of FMRP.

Here we investigate the behavioral and cellular changes induced by FMRP knockdown in the optic tectum. We use translation-blocking antisense morpholino oligonucleotides to decrease FMRP expression in the optic tectum during a key developmental period. We measured behavior using several assays that measure swimming speed, escape responses, social aggregation, and seizure susceptibility. We also investigated the electrophysiological properties of cells in the optic tectum. Our results show that FMRP knockdown results in decreased swimming speed, reduced schooling behavior and decreased seizure severity. However, FMRP knockdown does not perturb intrinsic properties of tectal neurons, but rather results in enhanced synaptic inhibition. This circuit abnormality is consistent with the behavioral results and shows that the early effects seen for FMRP knockdown have important functional consequences.

## Methods

All animal experiments were performed in accordance with and approved by Brown University Institutional Animal Care and Use Committee standards.

### Experimental animals

Tadpoles were raised in Steinberg’s rearing media on a 12 h light/dark cycle at 18–21 °C for 7–8 d, until they reached developmental stage 46 [10, 14]. They were then electroporated with the FMR1 or scrambled control morpholino and reared until they reached developmental stages of either 47 or 49, depending on the experiment (see below). Developmental stages of tadpoles were determined according to [14]. The rearing medium was renewed every 3 d. Tadpoles that were used for acoustic startle, schooling, or seizure protocols were not used again for other experiments, whereas after visual avoidance experiments, tadpoles were in some cases also used in startle and schooling experiments after having 24 h of rest in the rearing solution. At least two different clutches of tadpoles from different husbandry were used for every set of behavioral experiments. Animals of either sex were used because at these developmental stages tadpoles of either sex are phenotypically indistinguishable.

### Morpholinos

As described and validated in a prior study [10], a Xenopus laevis homolog of FMR1, fmr1a, was knocked down using a 3′ lissamine-tagged translation-blocking antisense morpholino oligonucleotide (GeneTools) with the sequence 5′- AGCTCCTCCATGTTGCGTCCGCACA-3′ (start codon underlined), referred to as fmr1a MO to generate FMRP knockdown (FMRP KD). Control lissamine-tagged oligonucleotides had the sequences 5′-TAACTCGCATCGTAGATTGACTAAA-3′ or 5′-CCTCTTACCTCAGTTACAATTTATA-3′, referred to as control. Morpholinos were dissolved in water. Morpholinos were injected into the brain ventricle, then platinum electrodes were placed on each side of the midbrain and voltage pulses were applied across the midbrain to electroporate optic tectum cells in stage 46 tadpoles.

### Behavioral experiments

#### Seizures

For seizure experiments, stage 47 tadpoles were transferred into individual wells in a six-well plate (Corning), each filled with 7 ml of 5 mM pentylenetetrazol (PTZ) solution in Steinberg’s rearing media. The plate was diffusely illuminated from below and imaged from above with a SCB 2001 color camera (Samsung) at 30 frames/s. Tadpole positions were tracked in Noldus EthoVision XT (Noldus Information Technology) and processed offline in a custom MATLAB program (MathWorks). Onset of regular seizures happened on average 3.9 ± 1.3 min into the recording; seizure events were defined as periods of rapid and irregular movement, interrupted by periods of immobility [15], and were detected automatically using swimming speed thresholding at a level of half of the maximal swimming speed. Frequency of seizures and length of seizure events were measured across 5 min intervals of a 20-min-long recording.

### Collision avoidance

Stage 49 tadpoles were placed in a clear plastic Petri dish (8.5 cm in diameter) filled to an approximate depth of 1 cm with Steinberg’s solution at 18 °C. The dish was put on top of a CRT monitor screen (maximum luminance, 57 cd/m^2^ and minimum luminance, 0.3 cd/m^2^; Dell Ultrascan 1600 SH Series; Dell Computer Company) and screened from all sides with an opaque black cloth. Stimuli were generated by a custom-written MATLAB program using the Psychophysics Toolbox [16, 17]. A black circle of a radius of 0.3 cm was projected in the center of the dish. Every 30 s, this circle was sent toward the tadpole at a speed of 1.4 cm/s. Only collisions in which the animal was swimming within 1 s before the encounter with the circle were included in the dataset. Experiments were performed in the morning (from 9:00 A.M. to 1:00 P.M.), because animals seemed to be less responsive in the afternoon; each testing session lasted for 5 min. Videos were acquired in EthoVision; both the tadpole and the stimulus were manually tracked offline, and trajectories were exported for additional automated analysis in MATLAB. Avoidance response initiation points were identified as points of peak acceleration immediately after an encounter with a visual stimulus; escape speed was averaged over a 17 ms window (five frames) around the swimming velocity peak.

### Schooling

Fifteen to twenty tadpoles at developmental stage 49 were transferred to a glass bowl 17 cm in diameter (for each batch, control tadpoles matched FMRP KD tadpoles in number). A still image of tadpole distribution in the bowl was made every 5 min using Yawcam software (Magnus Lundvall, Yawcam) for 1 h (13 images per experiment). A strong acoustic stimulus was delivered 2.5 min after each photo was taken to elicit a startle response and force tadpoles to redistribute [18]. Coordinates of tadpole heads and tails were tracked manually in NIH ImageJ and exported for additional processing in MATLAB. We defined neighboring tadpoles through point set triangulation and used a Kolmogorov–Smirnov test to compare distributions of inter-tadpole distances between FMRP KD tadpoles and matched controls. For all pairs of “neighboring tadpoles” that were located closer than 5.7 cm to each other (two-thirds of the bowl radius), we also estimated the angle between their orientations in the bowl [19, 20].

### Statistics and presentation of behavior data

For behavioral data, averages and SDs are presented. When the Mann–Whitney test was used to compare values between the groups, significance values were reported as P_MW_, whereas for Kolmogorov–Smirnov test, *p* values are reported as P_KS_. Sample sizes are reported as *n* = x, *N* = y, where lowercase n stands for the number of measurements and capital N stands for the number of animals.

### Electrophysiology experiments

For whole-brain recordings, tadpole brains were prepared as described by [21] and [22]. In brief, tadpoles were anesthetized in 0.02 % tricainemethane sulfonate (MS-222). To access the ventral surface of the tectum, brains were filleted along the dorsal midline and dissected in HEPES-buffered extracellular saline [in mM: 115 NaCl, 2 KCl, 3 Cacl_2_, 3 MgCl_2_, 5 HEPES, 10 glucose, and 0.1 picrotoxin, pH 7.2 (osmolarity 255 mOsm)]. Brains were then pinned to a submerged block of Sylgard in a recording chamber and maintained at room temperature (24 °C). To access tectal cells, the ventricular membrane surrounding the tectum was carefully removed using a broken glass pipette. For evoked synaptic response experiments, a bipolar stimulating electrode (FHC) was placed on the optic chiasm to activate retinal ganglion cell (RGC) axons.

Whole-cell voltage-clamp and current-clamp recordings were performed using glass micropipettes (8–12 MΩ) filled with K-gluconate intracellular saline [in mM: 100 K-gluconate, 8 KCl, 5 NaCl, 1.5 MgCl2, 20 HEPES, 10 EGTA, 2 ATP, and 0.3 GTP, pH 7.2 (osmolarity 255 mOsm)]. Recordings were restricted consistently to retinorecipient neurons in the middle one-third of the tectum, thus avoiding any developmental variability existing along the rostrocaudal axis [21, 23, 24]. Electrical signals were measured with a Multiclamp 700B amplifier (Molecular Devices), digitized at 10 kHz using a Digidata 1440A analog-to-digital board, and acquired using pClamp 10 software. Leak subtraction was done in real time using the acquisition software. Membrane potential in the figures was not adjusted to compensate for a predicted 12 mV liquid junction potential. Data were analyzed using AxographX software. The GABA_A_ antagonist picrotoxin (100 μM) was added to the external saline in a subset of experiments. Spontaneous synaptic events were collected and quantified using a variable amplitude template [25]. Spontaneous excitatory post-synaptic currents (sEPSCs) were recorded at −60 mV in the presence of picrotoxin, whereas spontaneous inhibitory post-synaptic currents (sIPSCs) were collected in control media at 5 mV (the reversal for glutamatergic currents). For each cell, 60 s of spontaneous activity was recorded. For evoked synaptic response experiments, a bipolar stimulating electrode (FHC) was placed on the optic chiasm to activate RGC axons. Synaptic stimulation experiments were conducted by collecting EPSCs evoked by stimulating the optic chiasm at a stimulus intensity that consistently evoked maximal amplitude EPSCs. Evoked responses at −45 mV (excitation) and 5 mV (inhibition) were used to calculate the excitation/inhibition (E/I) ratio. Excitation and inhibition were calculated as a measure of area under the curve for a 250 ms time window beginning at the onset of the synaptic response. Evoked monosynaptic events (at a stimulus intensity that does not evoke polysynaptic activity, typically 30–60 % of the maximum) were used to collect AMPA/NMDA ratios. Peak current amplitude at −65 mV (1 ms window at peak; 10–15 trials per cell) was used to calculate AMPAR-mediated currents, and average current amplitude collected at 55 mV (10 ms window 20 ms after peak AMPA; 5–15 trials per cell) was used to calculate NMDAR-mediated currents. Experiments to measure polysynaptic network activity were performed by collecting EPSCs evoked by stimulating the optic chiasm at a stimulus intensity that evoked the maximal amplitude EPSC. Quantification of polysynaptic activity was calculated by measuring the total change in current over 100 ms time bins beginning at the onset of the evoked response. A spontaneous barrage was defined as a change in holding current of 10 or 20 pA intervals for a period of >200 ms. To quantify intrinsic cell excitability, cells were presented with a series of depolarizing steps (20 pA intervals) in current clamp, starting from −65 mV. The number of spikes elicited by current injection was quantified using the following criteria: to qualify as a spike, the height of the spike had to be at least half the height of its preceding spike and no wider than three times the width of the first original spike [26]. Voltage-gated Na^+^ and K^+^ current–voltage (*I–V*) curves were calculated as in the study by [22], by measuring the early Na^+^ peak current and the steady-state K^+^ current. All data were tested for normality; parametric statistical tests were completed on normally distributed data and nonparametric Mann–Whitney *U* tests were completed on non-normal data. Graphs show mean and standard deviation as error bars, and data in the text show means and standard deviations, unless otherwise indicated.

## Results

Behavioral and electrophysiological experiments were performed on stage 49 tadpoles in which FMRP expression was knocked down using a morpholino-antisense oligomer [10] during critical neural proliferation and circuit wiring time periods, referred to as FMRP KD tadpoles throughout. Tadpoles were compared with a control group which was transfected with a scrambled version of the morpholino. Our results show behavioral and electrophysiological deficits that implicate impaired inhibitory circuitry as the primary change resulting from knockdown of FMRP.

**Fig. 1:**
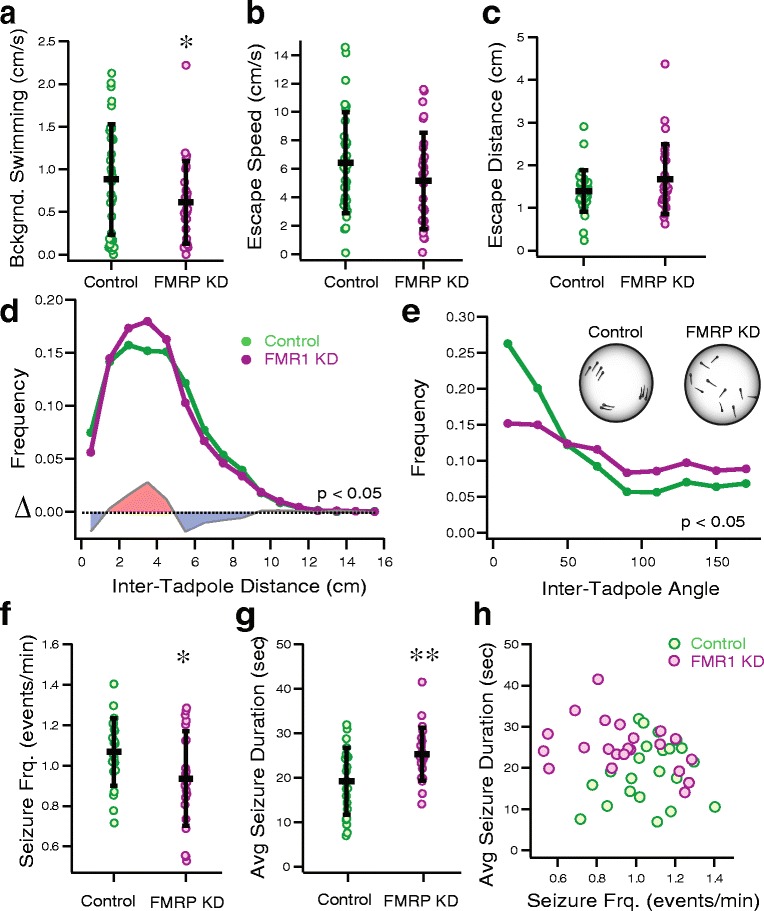
Background swimming speeds, schooling behavior and seizure severity are affected by FMRP KD. **a** Background swimming is decreased in FMRP KD tadpoles (P_t_ < 0.05). **b**–**c** Visual avoidance behavior is unaffected. Collision-escape velocity (**b**, the velocity of the tadpole after collision with virtual object) and escape distance (**c**, the distance between the tadpole and the virtual object when the tadpole initiates avoidance response) are unaffected (P_t_ > 0.05 for controls and FMRP KD tadpoles in **b**, **c**). **d**–**e** FMRP KD tadpoles show reduced schooling. **d** FMRP KD tadpoles have fewer long and short distances and more medium distances between neighboring tadpoles, indicating more dispersed swimming and decreased aggregation (*P*_*KS*_ < 0.05). **e** Control tadpoles have a higher frequency of less than 90° angles for co-orientation whereas FMRP KD tadpoles have no preference for alignment (*P*_*KS*_ < 10^−20^), inset. Diagram explaining schooling behavior, with small clusters of tadpoles with more short (inter-cluster) and long (intra-cluster) distances in controls, and more medium distances in FMRP KD tadpoles. Tadpoles are also co oriented with their nearest neighbor. **f**–**h** FMRP KD tadpoles seize significantly less frequently and for longer than controls, indicating decreased seizure susceptibility. **f** FMRP KD tadpoles seize with significantly reduced frequency compared to control MO tadpoles (*P*_*t*_ < 0.01), **g** FMRP KD tadpoles have significantly longer seizures (*P*_*t*_ < 0.005), **h** Seizure length plotted against seizure frequency indicates a negative correlation between the two, and separation between experimental groups

We performed a series of behavioral experiments designed to test various functional aspects of neural circuit development [20]. Visual avoidance behavior is a test useful for assaying basic swimming ability as well as overall visual system function by measuring escape responses in response to a virtual object [13]. The collision avoidance response is a conserved behavior in *Xenopus laevis* tadpoles, and across species that relies heavily on the optic tectum for sensory integration and perception. Organisms engage in this behavior to maneuver away from impending predators or objects. We observed that FMRP KD tadpoles have significantly decreased background swimming speed prior to stimulus presentation (Fig. 1a, P_t_ = 0.049, 0.91 cm/s ± 0.65, *N* = 6, *n* = 31 for control tadpoles; 0.61 cm ± 0.48, *N* = 6, *n* = 27 for FMRP KD tadpoles), indicating an overall decrease in activity levels. However, we found that FMRP KD tadpole collision escape responses were not significantly different from controls in measures of escape speed (Fig. 1b P_t_ > 0.089; 6.7 ± 3.5 cm/s, for control tadpoles; 5.1 ± 3.4 cm/s for FMRP KD tadpoles) and collision escape distance (Fig. 1c, P_MW_ = 0.777, 1.46 ± 0.4 cm for control tadpoles; 1.57 ± 0.8 cm for FMRP KD tadpoles). These data indicate that while FMRP KD tadpoles are overall slower swimmers, the basic visual function and escape responses are largely unchanged by FMRP KD.

Without major effects to the basic escape behavior, we wanted to identify if behaviors particularly relevant to FXS were affected in our FMRP KD tadpoles. A common behavioral marker of FXS and autism spectrum disorders (ASDs) is social interaction deficits. Tadpoles normally engage in a social aggregation behavior, called schooling [19]. Schooling is defined as structured aquatic animal aggregation marked by coordinated unidirectional group swimming behavior [27]. Recent work in our lab found that control tadpoles that are in schools normally swim parallel to each other (with an inter-tadpole body-axis angle less than 45°) and within at least two-thirds of the bowl’s radius to each other [20]. This schooling behavior requires integration of various sensory cues, including visual, auditory and olfactory. Here, we observed abnormal schooling patterns in FMRP KD tadpoles (Fig. 1d–e). Comparing the distributions of angles and distances of 2035 control and 2054 FMRP KD sample measurements in 51 experimental runs, we found that FMRP KD tadpoles showed a significantly different distribution of inter-tadpole distances (Fig. 1d, P_KS_ < 0.05; *N* = 51 for FMRP and control tadpoles; see Fig. 1e inset), with decreased short and long inter-tadpole distances and increased intermediate distances, indicating more disperse swimming and less aggregation in FMRP KD tadpoles. Consistently, FMRP KD tadpoles also had fewer neighboring tadpoles that swam in the same direction (angle < 45°) and more tadpoles that swam perpendicular and opposite (90° and 180°, respectively) to their neighboring tadpoles (Fig. 1e, P_KS_ < 10^−20^; *N* = 51 for both FMRP KD and control tadpoles). Both of these measures indicate that FMRP KD tadpoles show decreased schooling behavior.

Next we tested for seizure susceptibility. When presented with a convulsant, tadpoles develop seizures within 20 min [15]. Prior work has shown that abnormalities in excitatory connectivity, or in local inhibition can strongly affect the severity and length of these seizures. Seizures were induced pharmacologically with 5 mM pentylenetetrazol (PTZ) applied to the rearing media [20]. Under control conditions, increasing the concentration of PTZ results in an increase in seizure frequency and a decrease in seizure length. We found that FMRP KD tadpoles had less frequent (Fig. 1f, P_t_ = 0.038 1.06 ± 0.16 events per minute for controls and 0.94 ± 0.23 events per minute for FMRP; *N* = 22 for controls and FMRP KD tadpoles) and significantly longer seizures (Fig. 1g, P_t_ < 0.005, length of seizure 19.24 ± 7.37 s for controls and 25.28 ± 5.86 s for FMRP). The decreased seizure frequency and increased seizure length (Fig. 1h) are consistent with decreased seizure severity, indicating that FMRP KD tadpoles show decreased seizure susceptibility.

Disrupted schooling behavior indicates abnormal integration of multisensory input potentially resulting from abnormal neural circuit development, and decreased seizure susceptibility and lower baseline swimming could indicate decreased overall excitation or enhanced inhibition in the brain. Together with the prior observation that FMRP KD tadpoles show changes in tectal neuron proliferation and arborization [10], these findings lead us to perform electrophysiological recordings to assess whether there was a corresponding alterations in tectal excitability or network function. We explored three potential mechanisms to account for decreased tectal activity, including lowered intrinsic excitability of tectal neurons, abnormal development of excitatory synaptic transmission and increased synaptic inhibition.

**Fig. 2:**
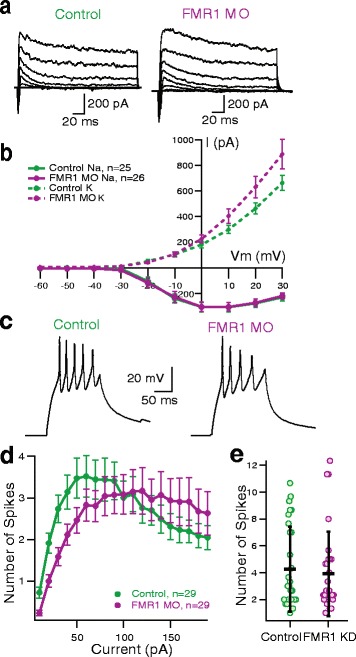
Intrinsic properties of tectal neurons are similar between control and FMRP KD tadpoles. **a** Example traces showing voltage gated inward and outward currents evoked by a series of depolarizing pulses. Voltage steps, 0–90 mV in 10 mV increments, were presented for 150 ms while current was measured, from a holding potential of −60 mV. **b** Current–voltage relationship of inward sodium (Na) and outward potassium (K) voltage-gated currents. Dotted lines are the K current, solid lines are Na current. **c** Example spiking traces. Current steps, 10-200pA in 10pA increments were presented for 150 ms while voltage was measured. Voltage injections of 40pA and 180pA shown. **d** Current vs. spiking relationship. *n* = 29 per group, At 10pA injection, *p* = 0.002 and at 20pA injection, *p* = 0.002 (multiple t-tests by current step, Sidak-Bonferroni multiple comparisons correction). **e** Maximum number of spikes at a given current injection. *n* = 29 per group, P_MW_ =0.8376

To begin to examine the underlying causes of the behavioral phenotypes, we first investigated the intrinsic properties of the tectal neurons [28]. We found no difference in membrane capacitance or action potential threshold, but membrane resistance was significantly lower in FMRP KD cells (Table 1). We also measured voltage-gated sodium (Fig. 2a, b, P_t_ = 0.86, Peak current: −333.1 ± 39.0 pA, *n* = 25 for controls, −331.4 ± 38.5 pA, *n* = 26 for FMRP KD) and potassium currents (Fig. 2a, b, P_t_ = 0.054, Max current: 664.5 ± 59.2 pA, *n* = 25 for controls, 889.3 ± 114.5 pA, *n* = 26 for FMRP KD), and found no significant difference. We also did not find any significant differences in spike output evoked by direct depolarization over a range of current injections (Fig. 2c–e) and the maximum spike count (Fig. 2d, 4.3 ± 3.2 spikes, *n* = 29 for controls, 3.9 ± 3.1 spikes, *n* = 29 for FMRP KD). These results indicate that despite altered arborization, FMRP KD does not affect normal development of intrinsic excitability in tectal neurons.

**Table 1:** Cell size, action potential threshold, and membrane resistance in control and FMR1 KD tadpoles

	Control	FMR1 KD
Membrane Capacitance	10.82 ± 4.90 pF, *n* = 31	11.28 ± 2.90 pF, *n* = 33
Membrane resistance^*^	2.419 ± 1.93 mΩ, *n* = 31	1.387 ± 0.638 mΩ, *n* = 33
Action potential threshold	−22.90 ± 4.55 mV, *n* = 29	−20.93 ± 3.80 mV, *n* = 29

*Membrane resistance is significantly different in FMR1 KD tadpoles (*p* = 0.0261, Mann–Whitney *U*-test). Mean ± standard deviation reported for each group

**Fig. 3:**
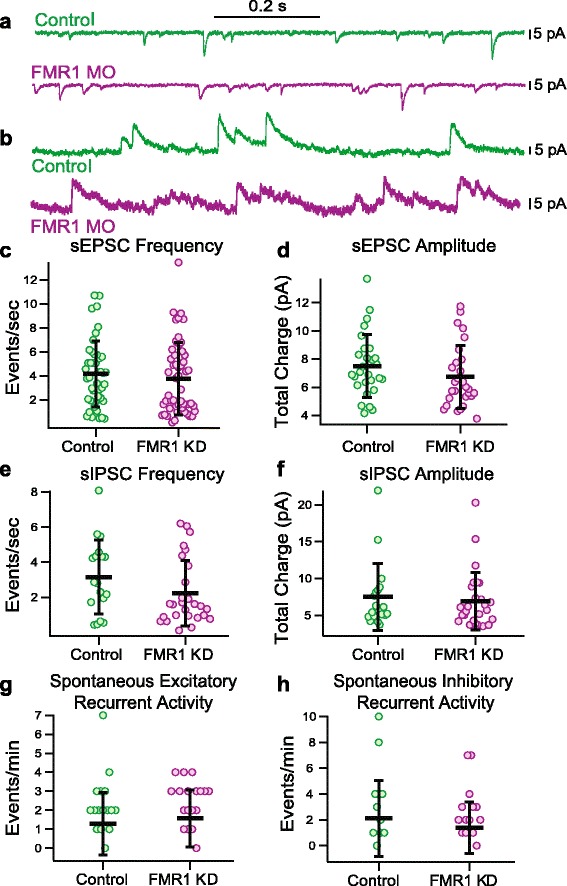
Spontaneous synaptic activity in tectal neurons is similar between control and FMRP KD tadpoles. **a** Example spontaneous EPSCs recorded at -60 mV in the presence of GABA_A_ receptor blocker, picrotoxin. **b** Example spontaneous IPSCs recorded at +5 mV, the reversal potential for excitatory currents. **c** Frequency of sEPSCs. *n* = 28 for each group, P_MW_ = 0.6784. **d** Amplitude of sEPSCs. *n* = 28 for each group, P_MW_ =0.1069. **e** Frequency of sIPSCs. *n* = 18 control, *n* = 27 FMRP KD, P_MW_ =0.1789. **f** Amplitude of sIPSCs. *n* = 18 control, *n* = 27 FMRP KD, P_MW_ =0.5740. **g** Excitatory recurrent activity, defined as the presence of a barrage of activity at least 200 ms in duration with a change in holding current of at least 10pA. *n* = 28 for each group, P_MW_ =0.3136. **h** Inhibitory recurrent activity, defined as the presence of a barrage of activity at least 200 ms in duration with a change in holding current of at least 10pA. *n* = 18 control, *n* = 27 FMRP KD, P_MW_ =0.5101

We next examined the connectivity of the tectal network. Given the known cell proliferation defect [10], we know that there are fewer cells in the tectum. Therefore, the network properties could be altered. However, we found no difference in the spontaneous synaptic activity of tectal cells (Fig. 3a, b). Between groups, spontaneous excitatory post-synaptic currents (sEPSC) had similar frequency (Fig. 3c, P_MW_ > 0.67, 4.73 ± 2.95 events per second, *n* = 28 for controls, 5.18 ± 3.24 events per second, *n* = 28 for FMRP KD) and amplitude (Fig. 3d, P_MW_ > 0.11, 7.50 ± 2.23 pA, *n* = 28 for controls, 6.73 ± 2.23 pA, *n* = 28 for FMRP KD), as did the inhibitory post-synaptic currents (sIPSC) (Fig. 3e, frequency: P_t_ = 0.13, 3.16 ± 2.12 events per second, *n* = 18 for controls, 2.23 ± 1.88 events per second, *n* = 27 for FMRP KD; Fig. 3f, amplitude: P_t_ =0.63, 7.51 ± 4.56 pA, *n* = 18 for controls, 6.90 ± 3.89 pA, *n* = 27 for FMRP KD). Spontaneous recurrent activity is measured as the occurrence of barrages of activity of at least 200 ms duration with a 10 pA change in holding current and indicates the amount of recurrent circuitry present in the network. It is a measure of how interconnected the tectal network is. We found no difference in the frequency of spontaneous recurrent activity (Fig. 3g, excitatory: P_t_ =0.48, 1.28 ± 1.65 events per minute, *n* = 29 for controls, 1.57 ± 1.50 events per minute, *n* = 28 for FMRP KD; Fig 3h, inhibitory: P_t_ =0.33, 2.11 ± 2.95 events per minute, *n* = 18 for controls, 1.39 ± 1.99 events per minute, *n* = 28 for FMRP KD). These results indicate that the synaptic connectivity of the cells present in the network appears similar, and that a given cell has roughly the same number, frequency and type of synapses.

**Fig. 4:**
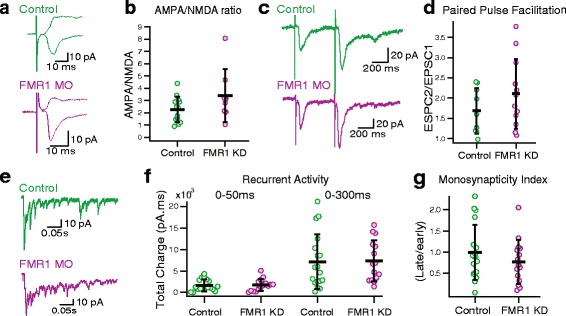
Excitatory evoked activity is not different in FMRP KD tadpoles. **a** Example traces of excitatory evoked activity, used to calculate the AMPA:NMDA ratio (top, control, average of 24 traces (−65 mV) and 15 traces (+55 mV)); bottom, FMRP KD, average of 19 traces (−65 mV) and 23 traces (+55 mV)). **b** The AMPA:NMDA ratio quantifies the size of the response at -65 mV and at +55 mV and is not different between groups. *n* = 14 (control), *n* = 9 (FMRP KD), P_t_ = 0.77. **c** Example traces of paired pulse facilitation (single paired stimuli) collected at a holding potential of −60 mV. **d** The ratio of the second pulse to the first pulse in a paired pulse protocol shows the level of facilitation at the synapse. *n* = 9 (control), *n* = 12 (FMRP KD), P_t_ =0.82. **e** Example traces of recurrent activity (single stimulation). **f** Recurrent activity quantified over two timeframes. 0-50 ms is primarily driven by the monosynaptic visual afferents while 0-300 ms primarily measures the polysynaptic activity evoked by local tectal networks. *n* = 18 (control), *n* = 16 (FMRP KD), P_MW_ =0.84 (0-50 ms), P_MW_ =0.91 (0-300 ms). **g** Monosynapticity, the ratio of the monosynpatic response (0-50 ms) to the polysynaptic response (100-200 ms). *n* = 18 (control), *n* = 15 (FMRP KD), P_MW_ =0.44

While spontaneous synaptic transmission might be indicative of the total input a tectal neuron receives, it does not tell us anything specific about the state of visual inputs to the tectum. To that end, we investigated responses to evoked visual stimulation. Evoked synaptic activity provides a measure of synaptic strength and network connectivity. First, we assayed basic synaptic properties. The AMPA to NMDA ratio is a measure of synaptic maturation, as cells incorporate additional AMPA receptors as they mature; therefore more mature cells have a larger AMPA:NMDA ratio [21]. We found no difference in the AMPA:NMDA ratios, indicating that FMRP KD does not affect synaptic maturation (Fig 4a, b, P_t_ = 0.78, 2.3 ± 1.0, *n* = 13 for controls, 3.4 ± 2.2, *n* = 8 for FMRP KD). Furthermore, paired pulse facilitation, a presynaptic measure of synaptic efficacy [29], was not affected by FMRP KD (Fig 4c, d, P_t_ > 0.22, 1.68 ± 0.60, *n* = 12 for control, 2.10 ± 0.86, *n* = 9 for FMRP KD). In the tectum, afferent visual input recruits a large amount of recurrent excitation [30, 31]. First, we measured recurrent activity in response to visual pathway stimulation to assess network connectivity and monosynaptic responses to assess direct synaptic connections. There was no difference in the response size of excitatory monosynaptic or recurrent activity as measured by calculating the total charge during the response (Fig. 4e, f, 0-50 ms: P_t_ = 0.84, 1620.9 ± 1342.9 pA.ms, *n* = 18 for controls, 1716.7 ± 1395.7 pA.ms, *n* = 15 for FMRP KD; 0-300 ms: P_t_ = 0.91, 7126.6 ± 6365.6 pA.ms, *n* = 18 for controls, 7339.9 ± 4784.7 pA.ms, *n* = 15 for FMRP KD), indicating an overall normal network level responses to visual inputs. We also measured the ratio of charge evoked by the monosynaptic afferent input, to the total amount of network activity. This provides us with a measure which we term monosynapticity index, which is a measure of how much network activity is evoked by visual input [28]. We found no difference in monosynapticity between experimental groups (Fig. 4g, P_MW_ = 0.44, 1.0 ± 0.64, *n* = 18 for controls, 0.77 ± 0.51, *n* = 15 for FMRP KD), indicating that excitatory network connectivity is unaffected. Together, these data show that excitatory synaptic activity and recurrent excitation are not changed by FMRP knockdown by FMR1 morpholino despite decreased proliferation and dendritic arborization.

**Fig. 5:**
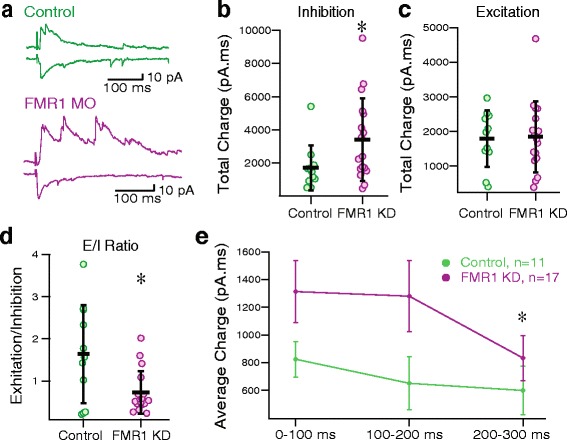
FMRP KD tadpole cells show increased evoked inhibitory activity. **a** Example traces of inhibitory evoked activity (recorded at +5 mv), and excitatory (recorded at −45 mv) used to calculate the excitation-inhibition balance (top, control; bottom, FMRP KD). **b** Evoked inhibitory recurrent activity at +5 mV, P_MW_ = 0.048, *n* = 11 (control), *n* = 17 (FMRP KD). **c** Evoked excitatory recurrent activity at -45 mV, *n* = 11 (control), *n* = 17 (FMRP KD). **d** Excitation to inhibition ratio, P_t_ = 0.009, *n* = 11 (control), *n* = 17 (FMRP KD). **e** Average charge of inhibitory responses calculated over bins of 100 msec following stimulus show altered time course of inhibitory responses in FMRP KD group

It has been noted in other studies that a discoordination of excitation and inhibition may underlie many neurodevelopmental disorders [32, 33]. To that end, we investigated evoked inhibitory recurrent synaptic activity. We found that evoked inhibitory currents were significantly greater in FMRP KD tadpoles (Fig. 5a, b, P_MW_ = 0.048, 1714 ± 1363 pA.ms, *n* = 11 for controls, 3421 ± 2490 pA.ms, *n* = 17 for FMRP KD). However, the excitation is not affected by FMRP KD (Fig. 5c, P_MW_ > 0.88, 1789 ± 817.5 pA.ms, *n* = 11 for control, 1845 ± 1029 pA.ms, *n* = 17 for FMRP KD), which yields a significantly smaller excitation to inhibition ratio in FMRP KD tadpoles (Fig. 5d, P_t_ = 0.009, 1.64 ± 1.17, *n* = 11 for controls, 0.73 ± 0.50, *n* = 17 for FMRP KD). We also looked at the overall time course of recurrent inhibition, and found that inhibitory activity remained elevated for a longer time following synaptic stimulation in the FMRP KD tadpoles (Fig. 5a, e, 0-100 ms: 824.6 ± 128.6 pA.ms for controls, 1313.1 ± 222.3 pA.ms for FMRP KD; 100-200 ms: 651.0 ± 191.5 pA.ms for controls, 1279.9 ± 255.8 pA.ms for FMRP KD; 200-300 ms: 599.1 ± 176.8 pA.ms for controls, 833.4 ± 163.0 pA.ms for FMRP KD; *n* = 11 for controls, *n* = 17 for FMRP KD), indicating altered dynamics of inhibitory circuits.

## Discussion

Our findings indicate that FMRP knockdown by FMR1 morpholino in the developing optic tectum has behavioral and electrophysiological consequences. Behaviorally, FMRP KD animals showed slightly reduced baseline swimming activity, decreased schooling behavior and decreased seizure susceptibility, but normal visual avoidance. These behavioral findings are consistent with decreased excitation within the tectum. Electrophysiologically we confirmed that neuronal intrinsic excitability and development of synaptic connectivity appear to be normal, however we found a significantly larger amount and longer lasting evoked network inhibition within the tectum, suggesting that this imbalance in the excitation to inhibition ratio may be responsible for the behavioral phenotypes.

These findings are unexpected since Faulkner et al. [10] identified a clear proliferative defect and abnormal tectal cell dendritic branching. Our experiments here show that despite these deficits individual cells develop normal excitability profiles and that excitatory and inhibitory synapses show normal maturation in the absence of FMRP. Nevertheless our findings also confirm that there is abnormal network connectivity since there is an increase in evoked inhibitory currents. Since we did not observe any differences in spontaneous inhibitory transmission, our findings suggest that the effects observed are likely occurring in cells other than the principal deep layer cells that we recorded from in this study. For example one could speculate that a change in intrinsic excitability of inhibitory interneurons, or increased excitatory drive to interneurons may explain our findings. It could also be that there are a greater number of inhibitory cells present in the tecta of FMRP KD tadpoles, or elevated numbers of excitatory interneurons driving inhibition. These changes could link our findings to the abnormal neuronal proliferation described previously using the same experimental manipulation. Furthermore, our findings that excitatory synapse maturation is unaffected, together with previous findings that neuronal proliferation is disrupted, are consistent with the view that FMRP deficits can have effects much earlier in development than previously thought. Development of normal brain connectivity requires a careful interplay between cell proliferation, migration and differentiation, and altering this interplay could result in abnormal neural circuit formation and behavioral deficits, even if individual neurons still appear to develop normally [34].

Disruptions in the normal balance of excitation and inhibition during development have been implicated in a number of neurodevelopmental disorders, ranging from schizophrenia to autism [33, 35–37]. Thus our findings are consistent with this view, and consistent with findings in other models of autism in which increased levels of inhibition are observed [38]. Other studies, in contrast, have also associated autism and Fragile X syndrome with alterations that result in decreased inhibition over excitation, resulting in increased seizure susceptibility [39–42]. For example in the rodent cortex, knockout of the FMR1 gene results in decreased synaptic drive to inhibitory interneurons and increased intrinsic excitability of excitatory cortical neurons, which result in prolonged evoked “up states” [43, 44]. In the amygdala, FMR1 knockouts have overall decreased tonic inhibition, leading to altered E/I balance and hyper excitability (Martin 2014). Interestingly, in the amygdala during development (p14) the FMR1 knockouts show a transient period of enhanced inhibition, which they ascribe to a homeostatic adaptation that ultimately fails in adult animals [41]. It is also worth noting that the seizures associated with Fragile X syndrome tend to be relatively mild and occur in only 10–20 % of individuals. This suggests that there could be a compensatory mechanism to counteract increased excitability, and perhaps this mechanism is more strongly evident in our brain structure and model organism. The effects of FMRP knockout also seem to be brain region specific and may manifest differently in the midbrain. However, the basic principle that small disruptions in network connectivity can lead to imbalances in information processing, which can then cascade into visible behavioral phenotypes, seems to be a common factor conserved across different brain regions, species and disorders.

To follow up on this study, it will be important to investigate the network properties of the optic tectum. The interaction of the decreased neural proliferation found by Faulkner et al. [10] and the increased evoked inhibition at the single cell level may manifest itself as a change in how the neural network interacts and interprets visual information to generate behavioral output. These studies could be carried out via in vivo Ca++ imaging of ensemble neuronal activity within the tectum [45]. It will also be important to identify whether the increased evoked inhibition is due to a larger proportion of GABAergic interneurons, or to alterations in interneuron physiology.

## Conclusions

Fragile X Syndrome and other neurodevelopmental disorders affect people in many ways, but have proven difficult to treat clinically. With our work, we have shown an explanation of why that may occur. It is clear that one particular insult does not result in a single, robust phenotype. Our research shows the opposite: small changes at the cellular level, combined with a neural proliferation defect, gives rise to the behavioral phenotype.

## Abbreviations

ASD, autism spectrum disorders; CNS, central nervous system; FMR1, fragile X mental retardation 1 gene; FMRP, fragile X mental retardation protein; FXS, Fragile X Syndrome; K, Potassium; KD, knock down; MO, morpholino; Na, sodium; Pks, *P* value for Kolmogorov-Smirnov test; Pmw, *P* value for Man-Whitney *u*-test; Pt, *P* value for *t*-test; PTZ, pentylenetetrazol; RGC, retinal ganglion cell; RNA, ribonucleic acid; sEPSC, spontaneous excitatory post-synaptic current; sIPSC, spontaneous inhibitory post-synaptic current
